# Synaptotagmin I delays the fast inactivation of Kv1.4 channel through interaction with its N-terminus

**DOI:** 10.1186/1756-6606-7-4

**Published:** 2014-01-14

**Authors:** Chunliang Xie, Haibo Su, Tianyao Guo, Yizhong Yan, Xiaozhen Peng, Rui Cao, Ying Wang, Ping Chen, Xianchun Wang, Songping Liang

**Affiliations:** 1 Key Laboratory of Protein Chemistry and Developmental Biology of Ministry of Education, College of Life Sciences, Hunan Normal University, Changsha, Hunan 410081, P. R. China; 2 Institute of Bast Fiber Crops, Chinese Academy of Agricultural Sciences, Changsha 410205, P. R. China

**Keywords:** Synaptotagmin I, Kv1.4, Interaction, Inactivation kinetics, Regulation

## Abstract

**Background:**

The voltage-gated potassium channel Kv1.4 is an important A-type potassium channel and modulates the excitability of neurons in central nervous system. Analysis of the interaction between Kv1.4 and its interacting proteins is helpful to elucidate the function and mechanism of the channel.

**Results:**

In the present research, synaptotagmin I was for the first time demonstrated to be an interacting protein of Kv1.4 and its interaction with Kv1.4 channel did not require the mediation of other synaptic proteins. Using patch-clamp technique, synaptotagmin I was found to delay the inactivation of Kv1.4 in HEK293T cells in a Ca^2+^-dependent manner, and this interaction was proven to have specificity. Mutagenesis experiments indicated that synaptotagmin I interacted with the N-terminus of Kv1.4 and thus delayed its N-type fast inactivation.

**Conclusion:**

These data suggest that synaptotagmin I is an interacting protein of Kv1.4 channel and, as a negative modulator, may play an important role in regulating neuronal excitability and synaptic efficacy.

## Background

Voltage-gated potassium (Kv) channels set the resting membrane potential and shape the action potential wave-form and frequency to tune excitability in the nervous system [1]. A-type Kv channels activate at subthreshold membrane potentials, inactivate rapidly with a time course of less than 100 ms, and rapidly recover from inactivation. In presynaptic terminals, A-type channel inactivation is responsible for frequency-dependent action potential broadening, a phenomenon that allows nerve terminals to regulate the neuronal excitability and synaptic transmission during high-frequency stimulation [2]. Kv1.4, a prominent member of the Kv1 family [3], is a fast inactivation A-type Kv channel [4,5] which is widely distributed in the central nervous system, where it is concentrated in axonal membranes or near axon [6], and also presents in the cardiac ventricular endocardium [7]. The up-regulation of Kv1.4 expression could lead to hypertrophy and heart failure, indicating that the behavior of this channel is of particular clinical importance [8,9].

Although Kv1.4 contributes to a presynaptic A–type current to regulate neurotransmitter release [10], how the Kv1.4 is regulated has not been fully understood. The native Kv1.4 channel often forms a tetramer by α subunits. However, the properties of Kv1.4 channel are modulated by a family of regulatory proteins in addition to the α subunits, such as Kvβ subunits that coassemble with the Kvα proteins through direct protein-protein interaction and affect the gating, permeability and pharmacology of the ion channel. Furthermore, the Kvβ subunits influence the number and subcellular localization of the ion channel in plasma membrane and synapse by promoting its trafficking and targeting processes [11]. In addition, intracellular signaling proteins can also modulate Kv1.4 channel function. For example, Kv1.4 channel is an *in vivo* substrate for CaMKII [12]. The phosphorylation of Kv1.4 by CaMKII can modulate the inactivation kinetics of this channel. It is suggested that kinases and phosphatases, as well as other signaling and scaffolding proteins, may be intimately associated with the ion channel in a regulatory protein complex [13]. Therefore, the functional complexity of Kv1.4 will increase as additional associated proteins are found.

In the present research, synaptotagmin I, a Ca^2+^ sensor playing a key role in the regulation of synaptic vesicle exocytosis [14], was shown by proteomic strategy to associate with Kv1.4 channel-containing complexes affinity purified from rat hippocampus. Further experiments revealed that there was specific and Ca^2+^-dependent interaction between synaptotagmin I and Kv1.4, and the interaction was not mediated by other synaptic proteins. Such specific interaction occurred between synaptotagmin I and the N-terminus of Kv1.4 channel and thus the fast N-type inactivation of the channel was delayed. The functional consequence of the interaction between synaptotagmin I and Kv1.4 is that the inactivation kinetics of Kv 1.4 is specifically modulated by synaptotagmin I, leading to the decrease in the neuronal excitability.

## Results

### Affinity purification and proteomic analysis of native Kv1.4 channel complex

**Figure 1:**
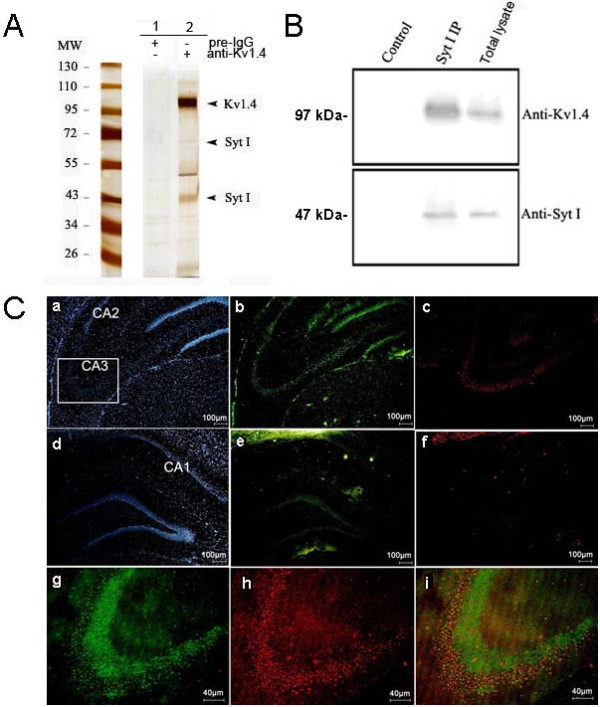
**Validation of interaction between synaptotagmin I and Kv1.4 channel. (A)** Silver-stained SDS-PAGE of the protein complexes affinity purified from rat hippocampal plasma membrane-enriched fraction either with a Kv1.4-specific antibody (anti-Kv1.4) or a preimmunisation IgG pool (Pre-IgG). Arrowheads denote the bands identified by nano-LC tandem mass spectrometry as Kv1.4 and synaptotagmin I, respectively. **(B)** Western blot showing reverse purification of Kv1.4 from the same fraction with anti-synaptotagmin I. **(C)** Immunohistochemical analysis of the colocalization of synaptotagmin I and Kv1.4 in rat hippocampus. (a)-(c) show the localizations of pyramidal cell nuclei, Kv1.4 and synaptotagmin I in the CA2- CA3 regions, respectively. (d)-(f) show the localizations of pyramidal cell nuclei, Kv1.4 and synaptotagmin I in the CA1 region and denote gyrus, respectively. (g)-(i) are the enlarged images of CA3 region showing the localizations of Kv1.4 (g), synaptotagmin I (h) and their colocalization (i) in this region.

Affinity purification and proteomic analysis were employed to identify the components of native Kv1.4 channel complex to find new modulatory factors of the ion channel. After the Kv1.4 channel complex was affinity purified with a Kv1.4-specific antibody (anti-Kv1.4) from plasma membrane-enriched protein fractions prepared from rat hippocampus, it was subjected to SDS-PAGE, using preimmunization immunoglobulins G (IgGs) as a negative control. The silver-stained protein bands obtained specifically with anti-Kv1.4 but not with preimmunization IgGs (Figure 1A) were selected for protein identification with CapLC-MS/MS. It was shown that the identified proteins included Kv1.1 protein, also an A-type Kv channel that had already been demonstrated to interact with Kv1.4 [15], several typical constituents of the synaptic exocytosis machinery (such as syntaxin1B, rab3A and synaptotagmin I) and Na^+^/K^+^-ATPase as well as cytoskeleton proteins (tubulin and actin) (Table 1). Synaptotagmin I was identified in the bands at about 47 kDa and 65 kDa (Figure 1A), suggesting that the protein exits in different forms due to the post-translation modification [16]. In view of the functional significance of synaptotagmin [14] and its abundant copurification with Kv1.4 using anti-Kv1.4 antibody (Figure 1A), the possible interaction between synaptotagmin I and Kv1.4 channel protein was further examined in subsequent investigations.

**Table 1:** Proteins affinity purified with anti-Kv1.4 from rat hippocampal plasma membranes-enriched protein fraction and identified by CapLC tandem mass spectrometry

**Categorization**	**Proteins**
Kv α subunits	Kv1.1, Kv1.4
Synaptic proteins	Syntaxin 1B, synaptotagmin I, synapsin, Rab3A, syntaxin-binding protein 1
Transporters	Na^+^/K^+^ ATPase (α, β)
Cytoskeleton proteins	Actin, tubulin

Procedures used for affinity purification and mass spectrometry as well as the criteria for protein identification are detailed in the Experimental Procedures.

The coassembly of synaptotagmin I and Kv1.4 channel complex was confirmed by subsequent reverse copurification from the rat hippocampal plasma membrane-enriched protein preparations with a synaptotagmin I -specific antibody (anti-synaptotagmin I). As illustrated by the western blot in Figure 1B, Kv1.4 was copurified by the anti-synaptotagmin I but not by the pool of preimmunization IgGs used as a control. These results further demonstrated that synaptotagmin I can associate with Kv1.4 channel complex.

### Overlapping expression profile of synaptotagmin I and Kv1.4 in hippocampus

Information regarding the distributions of synaptotagmin I and Kv1.4 in neurons is helpful to analyze their interaction. We performed dual labeling experiments to determine whether synaptotagmin I and Kv1.4 are colocalized in hippocampal neurons, as would be predicted if the interactions reported here are of physiological relevance. The expression profiles of synaptotagmin I and Kv1.4 in the hippocampus were analyzed by immunohistochemistry on adult rat brain sections and the specificity of the staining was confirmed by the preimmunization serum. The results in Figure 1C show that in the hippocampus Kv1.4 is predominantly expressed in the pyramidal cells of CA1-CA3 regions and the molecular layers of the dentate gyrus (b and e in Figure 1C), while synaptotagmin I staining is predominantly seen in the CA3 pyramidal cells but not the molecular layers of the dentate gyrus (c and f in Figure 1C). Both proteins are colocalized in the CA3 pyramidal cells (g to i in Figure 1C). These data demonstrate that synaptotagmin I has a narrower distribution range and its coexpression with Kv1.4 channel in CA3 pyramidal cells implies their potential physical relevance in the neurons.

### Interaction between synaptotagmin I and Kv1.4 coexpressed in HEK293T cells

**Figure 2:**
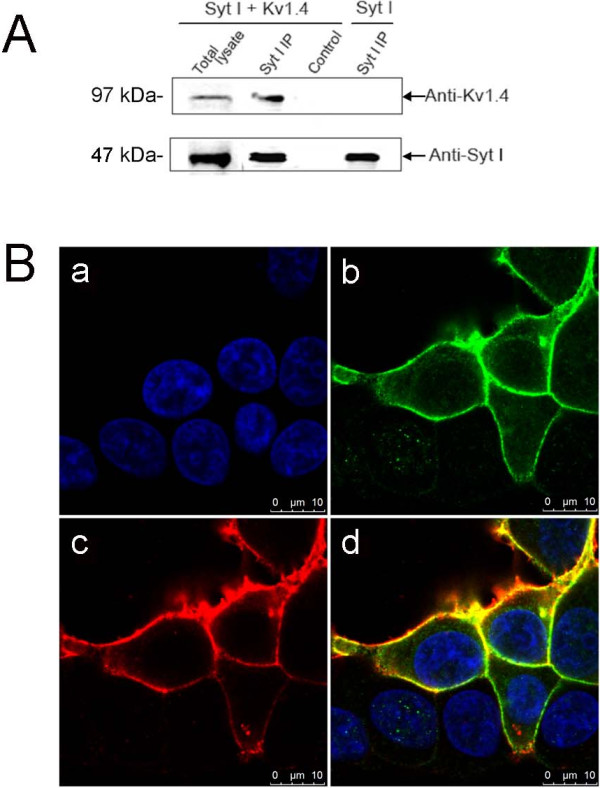
**Synaptotamin I interacts with Kv1.4 coexpressed in HEK 293 T cells. (A)** Recombinant full-length synaptotagmin I was expressed alone or coexpressed with Kv1.4 in HEK293T cells, and immunoprecipitations were performed using extract from the transfected cells. Probe of precipitated proteins with western blot indicated that Kv1.4 was coprecipitated with a polyclonal antibody against synaptotagmin I. Syt I, synaptotagmin I. **(B)** Subcellular localization of synaptotagmin I and Kv1.4 in HEK293T cells. Cells were incubated with Hochest 33342 (a) and transiently transfected with Kv1.4 cDNA (b), synaptotagmin I cDNA (c), and the combinations of Kv1.4 and synaptotagmin I cDNAs (d), respectively.

Although affinity purification and proteomic analysis suggested that synaptotagmin I could interact with Kv1.4 channel complex, we were not sure whether the interaction between synaptotagmin I and Kv1.4 was mediated by other protein(s) especially the synaptic proteins. To test the authenticity of the interaction between the two proteins, recombinant full-length synaptotagmin I and Kv1.4 were expressed in HEK293T cells. Immunoprecipitations with an antibody against synaptotagmin I (or with nonspecific control IgGs) were performed from cotransfected cell extract. The precipitates were tested for the presence of synaptotagmin I and Kv1.4 by western blot. The results (Figure 2A) showed that Kv1.4 was efficiently coprecipitated with the antibody against synaptotagmin I. At the same time, an immunofluorescence analysis on the HEK293T cells cotransfected with synaptotagmin I and Kv1.4 cDNAs was carried out. The results (Figure 2B) showed that both proteins were colocalized in the plasma membrane of HEK293T cells. These data demonstrated that synaptotagmin I and Kv1.4 have co-localization in the plasma membrane and the interaction between them does not require the mediation of other synaptic proteins.

### Synaptotagmin I modulates the inactivation kinetics of Kv1.4 in HEK293T cells

**Figure 3:**
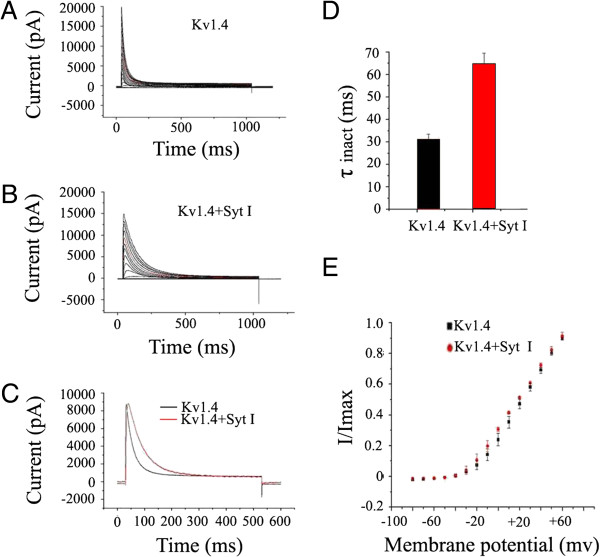
**Effects of synaptotagmin I on Kv1.4 kinetics expressed in HEK293T cells. (A)** Representative traces of Kv1.4 currents expressed alone. **(B)** Representative traces of Kv1.4 currents coexpressed with synaptotagmin I. Currents were elicited by depolarizing steps from -80 to 70 mV in 10 mV increments every 45 s from a holding potential of -80 mV. **(C)** Currents recorded upon depolarization to +30 mV. **(D)** Inactivation time constants (τ_inact_) of the indicated channel obtained at +30 mV. Data are expressed as mean ± SD. The τ_inact_ of Kv1.4 current expressed alone was 31.1 ms ± 2.3 ms (n = 13), compared with 64.8 ± 4.5 ms (n = 9) for Kv1.4 current coexpressed with synaptotagmin I (p < 0.05). **(E)** Activation curves of Kv1.4 and Kv1.4/synaptotagmin I. Current amplitudes recorded from the channels were normalized to the maximal response at +30 mV and plotted as a function of the membrane potential.

Given the overlapping in expression pattern and the importance of the two proteins in neurotransmission, the impact of the synaptotagmin I protein on Kv1.4 channel gating in HEK293T cells was further investigated with whole-cell patch-clamp technique. Figure 3A shows the rapidly inactivating A-type K^+^ currents recorded in response to depolarizing voltage steps (from -80 mV to +70 mV, 10 mV increment) in the whole-cell patch-clamp experiments with HEK293T cells that coexpressed Kv1.4 and EGFP. The decay of these A currents was adequately fit with a single exponential yielding a voltage-dependent inactivation time constant (τ_inact_) of 31.1 ± 2.3 ms (n = 13) at a membrane potential of +30 mV (Figure 3C and 3D). Coexpression of synaptotamin I and Kv1.4 markedly slowed the inactivation of Kv1.4 channel, with τ_inact_ of 64.8 ± 4.5 ms (n = 9) at +30 mV, increased by more than 2-fold (Figure 3B- 3D). In contrast, voltage-dependent activation of the channels was unaffected by the coexpressed synaptotagmin I (Figure 3E), suggesting that synaptotamin I only affects the inactivation kinetics of Kv1.4 channel.

### Modulation of Kv1.4 inactivation by synaptotagmin I requires intracellular Ca^2+^

**Figure 4:**
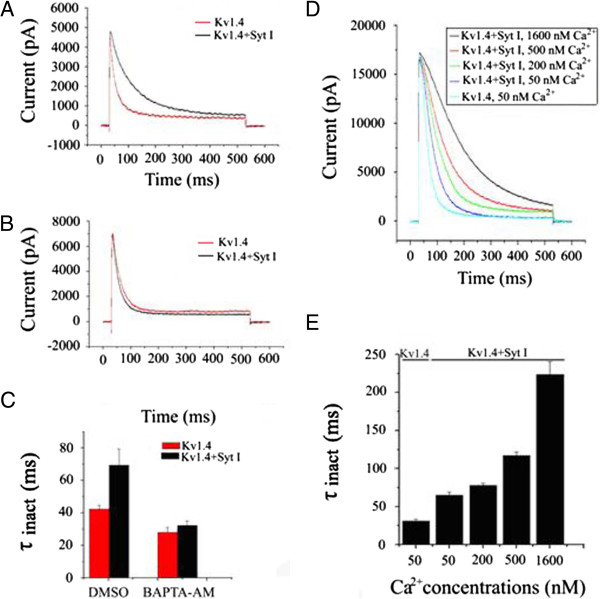
**Intracellular Ca**^**2+ **^**is required for the effect of synaptotagmin I on Kv1.4 currents.** One day after transfection, HEK293T cells were incubated for 20–26 h with BAPTA-AM (10 μM in 0.1%DMSO) or 0.1%DMSO alone as a control. Currents were measured at +30 mV 2 days after the injection. **(A)-(B)** Current traces of Kv1.4 and Kv1.4/synaptotagmin I after the cells were incubated with 0.1% DMSO **(A)** or 10 μM BAPTA-AM **(B)**. **(C)** Inactivation time constants (τ_inact_) under the two different conditions. Data are expressed as mean ± SD. After the cells were incubated with 0.1% DMSO, the τ_inact_ of Kv1.4 current expressed with and without synaptotagmin I were 38.1 ± 2.3 ms (n = 9) and 68.8 ± 2.6 ms (n = 9), respectively (p < 0.05). When the cells were incubated with BAPTA-AM, the τ_inact_ values of the channel under the two different conditions were not significantly different (p > 0.05). **(D)-(E)** Effects of synaptotagmin I on the inactivation of Kv1.4 channels at different concentrations of Ca^2+^**(D)** and their corresponding τ_inact_ values **(E)**.

Considering that synaptotagmin I takes part in the neurotransmission process as a Ca^2+^ sensor [17], we examined whether manipulation of cytosolic Ca^2+^ might influence the effects that synaptotagmin I had on Kv1.4 currents. After Kv1.4 cDNA was expressed alone or coexpressed with synaptotagmin I cDNA in HEK293T cells, the cells were incubated for a period of 20–26 h with the membrane-permeable Ca^2+^ chelator, BAPTA-AM (10 μM in 0.1%DMSO), or only with the solvent 0.1% DMSO as a control. The incubation with BAPTA-AM was started 1-day after transfection, when protein synthesis or trafficking was expected to occur at high rates. As a result, the inactivation of Kv1.4 was found to be delayed by the co-expressed synaptotagmin I when the HEK293T cells were incubated with solvent DMSO (Figure 4A) and the τ_inact_ values of Kv1.4 channel expressed alone and co-expressed with synaptotagmin I were 38.1 ± 2.3 ms (n = 9) and 68.8 ± 2.6 ms (n = 9) at a membrane potential of +30 mV, respectively (p < 0.05) (Figure 4C). In contrast, the effect of synaptotagmin I on Kv1.4 inactivation was prevented when HEK293T cells were incubated with the BAPTA-AM (Figure 4B) and the τ_inact_ values of the channel under the two different conditions were not significantly different (P > 0.05) (Figure 4C). These results demonstrated that modulation of Kv1.4 inactivation by synaptotagmin I requires intracellular Ca^2+^.

In order to further probe into the requirement for calcium ions, we used patch-clamp technique on HEK293T cells to investigate the effect of different concentrations of free calcium on the slowed inactivation of Kv1.4 caused by synaptotagmin I. To obtain specific free Ca^2+^ concentrations, 1 mM EGTA was added to the bath solution, and different concentrations of CaCl_2_ were added as calculated with the CABUF program. The results showed that, as the concentrations of added free calcium ions were increased from 50 nM to 1600 nM, the inactivation traces of Kv1.4 was shifted to the right accordingly. For example, at a free calcium ion concentration of 50 nM, Kv1.4 current inactivated rapidly, with a τ_inact_ value of 64.8 ± 4.5 ms (n = 10) at +30 mV; Upon application of 1600 nM free calcium ions, the inactivation of Kv1.4 was greatly slowed, resulting in a τ_inact_ value of 223.6 ± 17.3 ms (n = 10) (Figure 4D and 4E). In addition, in the low concentration range from 50 nM to 500 nM, there was a nearly linear increase in the τ_inact_ values (Figure 4E). These data showed that the effect of Ca^2+^ on the delay of Kv1.4 channel inactivation caused by synaptotagmin I is dose-dependent.

### Synaptotagmin I specifically modulates Kv1.4 channel currents

**Figure 5:**
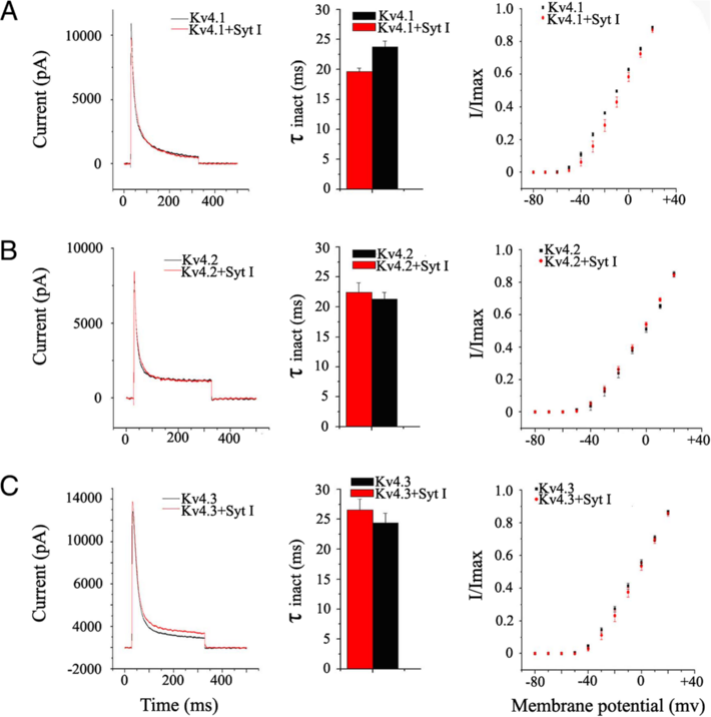
**Effects of synaptotagmin I on the kinetics of Kv4 family subunits expressed in HEK293T cells. (A)** Representative current traces of Kv4.1 channel and kv4.1/synaptotagmin I (left), their averaged inactivation time constants (τ_inact_) (middle) and their activation curves; Current amplitudes recorded were normalized to the maximal responses at +30 mV and plotted as a function of the membrane potential (right). **(B)** Representative current traces of Kv4.2 channel and kv4.2/synaptotagmin I (left), their averaged τ_inact_ (middle) and their activation curves; Current amplitudes recorded were normalized to the maximal responses at +30 mV and plotted as a function of the membrane potential (right). **(C)** Representative current traces of Kv4.3 channel and Kv4.3/synaptotagmin I (left), their averaged τ_inact_ (middle) and their activation curves; Current amplitudes recorded were normalized to the maximal responses at +30 mV and plotted as a function of the membrane potential (right).

In order to investigate the specificity for the modulation of Kv1.4 channel by synaptotagmin I, we examined whether synaptotagmin I also influenced other Kv channels. We chose Kv4.1, Kv4.2 and Kv4.3 of Kv4 family as experimental materials, because all of them and Kv1.4 express A-type currents and have similar inactivation mechanisms [18]. After the Kv4 plasmids were separately transfected alone or cotransfected with synaptotagmin I plasmid in HEK293T cells, the Kv channel kinetics were examined with whole-cell patch-clamp technique. Figure 5A shows the effect of synaptotagmin I on Kv4.1 channel. It could be found from the representative Kv4.1 current traces at +30 mV (*left*) that two current curves in the presence or absence of synaptotagmin I were very close to each other, with the τ_inact_ (*middle*) and activation current amplitude (*right*) values being not significantly different (P > 0.05). Likewise, Kv4.2 and Kv4.3 channels were not significantly affected by the coexpressed synaptotagmin I (Figure 5B and 5C). These results demonstrated that the interaction between synaptotagmin I and Kv1.4 is not a general feature of A-type potassium channels and the modulation of Kv1.4 by synaptotagmin I has specificity.

### Kv1.4 N-terminus is responsible for the interaction with synaptotagmin I

**Figure 6:**
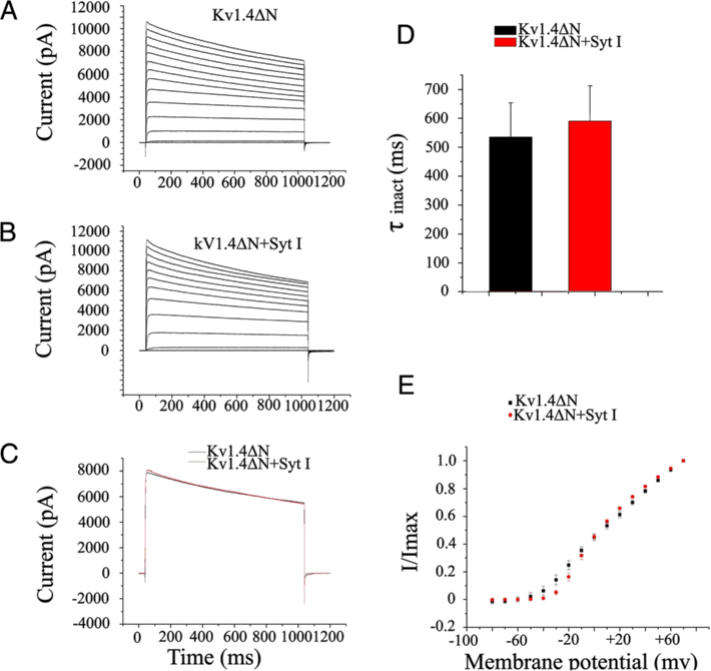
**Effect of synaptotagmin I on the kinetics of Kv1.4ΔN. (A)** Representative traces of Kv1.4ΔN currents expressed alone. **(B)** Representative traces of Kv1.4ΔN currents coexpressed with synaptotagmin I. Currents were elicited by depolarizing steps from -80 to +70 mV in 10 mV increments every 45 s from a holding potential of -80 mV. **(C)** Currents recorded upon depolarization to +30 mV. **(D)** Inactivation time constants (τ_inact_) of the indicated channel obtained at +30 mV. Data are mean ± SD of nine experiments. **(E)** Activation curves of Kv1.4ΔN and Kv1.4ΔN/synaptotagmin I. Current amplitudes recorded were normalized to the maximal response at +30 mV and plotted as a function of the membrane potential.

**Figure 7:**
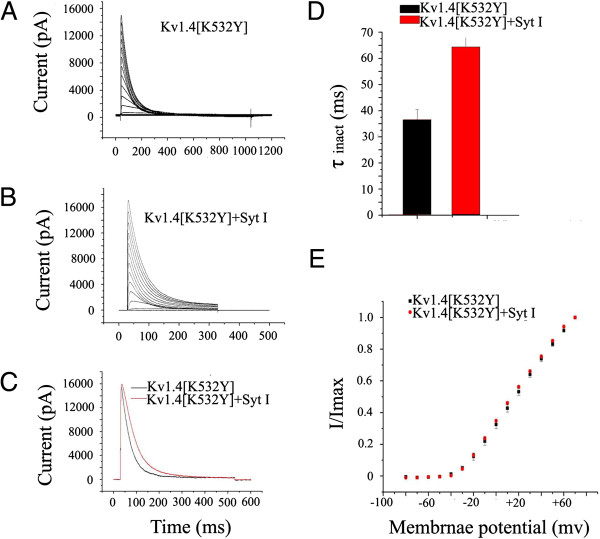
**Effect of synaptotagmin I on the kinetics of Kv1.4[K532Y]. (A)** Representative traces of Kv1.4[K532Y] currents expressed alone. **(B)** Representative traces of Kv1.4[K532Y] currents coexpressed with synaptotagmin I. Currents were elicited by depolarizing steps from -80 to +70 mV in 10 mV increments every 45 s from a holding potential of -80 mV. **(C)** Currents recorded upon depolarization to +30 mV. **(D)** Inactivation time constants (τ_inact_) of the indicated channels obtained at +30 mV. Data are expressed as mean ± SD. The inactivation of Kv1.4 [K532Y] was significantly (p < 0.05) affected by the coexpressed synaptotagmin I. The τ_inact_ of Kv1.4 [K532Y] was 36.6 ± 1.3 ms (n = 9). When coexpressed with synaptotamin I, its τ_inact_ was 64.5 ± 1.6 ms (n = 12). **(E)** Activation curves of Kv1.4[K532Y] and Kv1.4[K532Y]/synaptotagmin I. Current amplitudes recorded were normalized to the maximal response at +30 mV and plotted as a function of the membrane potential.

Kv1.4 channel has been demonstrated to exhibit slow C– and fast N-type inactivation, the latter incorporating an N-terminal “ball and chain” motif on the α-subunit to block the channel pore [19]. To be certain whether synaptotagmin I affects C–type or N-type inactivation of Kv1.4, two Kv1.4 cDNA mutants were constructed: Kv1.4ΔN and Kv1.4[K532Y]. Kv1.4ΔN lost N-type inactivation due to the deletion of amino acid residues 2–146 but retained C-type inactivation. Kv1.4[K532Y] had N-type inactivation but limited C-type inactivation due to a K-to-Y mutation [5]. The effects of synaptotagmin I on Kv1.4ΔN currents are shown in Figure 6. It can be seen that both of the activation and inactivation of Kv1.4ΔN mutant were not significantly affected by the coexpressed synaptotagmin I (Figure 6B- 6E). However, in view of the huge delay of inactivation in Kv1.4ΔN mutant itself (Figure 6A), it is difficult to expect to detect a further delay by the coexpression of synaptotagmin I. Therefore, it could not be concluded based on the results that the C-type inactivation of Kv1.4 was not affected by synaptotagmin I (see Discussion). In contrast, as shown in Figure 7A and 7B, the inactivation of Kv1.4 [K532Y] was markedly delayed by the coexpressed synaptotagmin I. At a membrane potential of +30 mV, the τ_inact_ of Kv1.4 [K532Y] was 36.6 ± 1.3 ms (n = 9), compared with 64.5 ± 1.6 ms (n = 12) when coexpressed with synaptotamin I, increased by nearly 2-fold. The difference was significant (p < 0.05) (Figure 7C and 7D). However, the activation of the channel was not significantly affected (Figure 7E). These results suggested that synaptotagmin I delays the fast N-type inactivation of Kv1.4 by interacting with the N-terminus of the channel.

## Discussion

### Association of synaptotagmin I with neuronal Kv 1.4 channel

The importance of synaptotagmins in vesicle exocytosis and other cellular activities has attracted considerable interest over the past decades of years. Synaptotagmins belong to a large family including at least 15 members [20] that display distinct expression patterns both in neuronal and non-neuronal tissues [21,22]. In nerve endings, synaptotagmins are predominantly localized in synaptic vesicles but also present in the presynaptic plasma membrane [23]. Synaptotagmin I is one of the major neuronal isoforms [24] and is so far the best characterized. A series of evidences indicate that synaptotagmin I is a multifunctional protein, interacting specifically with the neuronal SNARE (soluble N-ethylmaleimide-sensitive factor attachment proteins receptor) proteins to participate in the regulation of vesicle exocytosis, and also displaying direct high affinity interaction with neuronal ion channels and many other proteins in Ca^2+^-dependent as well as Ca^2+^-independent manners, thus modulating their physiological functions [14,25,26].

In neurons and neuroendocrine cells, regulated release of neurotransmitters, neuropeptides, and hormones relies on Ca^2+^-evoked exocytosis [27]. The three SNARE proteins, syntaxin, SNAP-25 and synaptobrevin constitute the minimal machinery for the exocytosis. Synaptotagmin I, acting as a Ca^2+^ sensor, can specifically interact with the SNARE machinery and play a critical role in SNARE-mediated membrane fusion. The close proximity of Ca^2+^ channels with synaptic proteins was demonstrated biochemically by co-immunoprecipitatin of N- and P/Q-type Ca^2+^ channels with syntaxin 1A and synaptotagmin [26,28-30]. At the active zone, where a large number of vesicles are packed close to clusters of Ca^2+^ channels, a physical association of synaptotagmin with the channels is almost inevitable [31]. A series of studies have demonstrated that the activity of Ca^2+^ channels is modified by syntaxin, synaptotagmin I and SNAP-25 alone, or in various combinations [31-33]. During the investigation of possible association of Na^+^ channels with synaptic proteins, Sampo et al. [34] found that synaptotagmin I displayed a direct high affinity interaction with neuronal voltage-sensitive Na^+^ channels, with the binding site on the cytosolic loop between domains I and II of the Na^+^ channel αIIA subunit. Moreover, the synaptotagmin/sodium channel complex was shown to be distinct from the synaptotagmin–SNARE complex that associates with voltage-sensitive Ca^2+^ channels. As regarding the relationship between synaptotagmins and Kv channels, Fili et al. [35] reported that there is a physical interaction in brain synaptosomes between Kvα1.1 and Kvβ subunits with syntaxin 1A occurring at least partially within the context of a macromolecular complex containing syntaxin, synaptotagmin and SNAP-25. However, the work of Fili et al. has not reported whether synaptotagmin interacts with the potassium channel subunits. To the best of our knowledge, up to now there have not been any reports on the interaction of synaptotagmins with K^+^ channels. In the present study, for the first time we discovered that synaptotagmin I can interact with Kv1.4 and the interaction does not require other synaptic proteins to mediate. Furthermore, synaptotagmin-Kv1.4 channel complex is similar to syntaxin-sodium channel complex [34] but distincts from the synaptotgamin-SNARE protein complex that associates with calcium channels [29,30].

### Features of synaptotagmin I interaction with Kv1.4 channel

As an A-type channel, Kv1.4 mediates rapidly inactivating outward rectifying currents and thus regulates membrane repolarization and thereby the electrical excitability [36,37]. The inactivation kinetics of the channel is modulated by many interacting proteins including those so called auxiliary subunits. Even minor alterations in the inactivation or recovery of the channel, such as those identified as the molecular basis of episodic ataxia [38] may result in considerable deleterious consequences. It has been reported that Kvβ1.1 can further potentiate the fast inactivation of Kv1.4 channel [39,40]. Jow et al. [41] also found a faster inactivation of Kv1.4 current when Kv1.4 and Kvβ1.1 were coexpressed in oocytes. Kvβ1.2 has been shown to increase the rate of inactivation of Kv1.4 and slow the rate of recovery from inactivation, and the mutation experiments indicated that Kvβ1.2C-terminus (Kvβ1.2-C) is responsible for interaction with the channel [42]. Co-expression of Kvβ3 with Kv1.4 shows that this β subunit can increase the rate of inactivation from 4- to 7-fold in the Kv1.4 channel [43]. When Kv1.4 is expressed with DPP10, a dipeptidyl peptidase-related ancillary subunit, the channel shows faster time to peak current and negative shift in the half-inactivation potential of steady-state activation and inactivation [18]. The inactivation kinetics of Kv1.4 is also modulated by protein phosphorylation. Roeper et al. [12] demonstrated that the inactivation gating of the Kv1.4 is controlled by CaMKII and the calcineurin/inhibitor-1 protein phosphatase cascade. In addition, the activation of Kv1.4 is also regulated by its degree of phosphorylation [44,45]. Some other proteins also display regulatory action. For example, the sigma receptor has been demonstrated to be a novel protein that mediates the modulation of Kv1.4 by psychotropic drugs through a unique transduction mechanism depending neither on G proteins nor protein phosphorylation [46].

In the present study, a new modulatory protein of Kv1.4, synaptotagmin I, was discovered. When we employed whole-cell patch-clamp technique to investigate the effect of synaptotagmin I on the properties of Kv1.4 channel gating in HEK293T cells. The results showed that, in control cells that only expressed Kv1.4 channel, the channel displayed rapidly inactivating A-type K^+^ currents in response to depolarizing voltage steps (Figure 3A), which is in agreement with the reported characteristics of Kv1.4 [4,5]. However, when Kv1.4 channel was coexpressed with synaptotamin I, the inactivation of the channel was markedly slowed, characterized by a more than 2-fold increase in the τ_inact_ value at a membrane potential of +30 mV, whereas the voltage-dependent activation of Kv1.4 channel was not obviously affected (Figure 3E). These results showed the first important feature of synaptotagmin I interaction with Kv1.4 channel: decreasing the inactivation rate of Kv1.4, without affecting its activation. That is to say, the action of synaptotagmin I on Kv1.4 is in contrast to those of the auxiliary subunit Kvβ1.1 and Kvβ3 as well as the dephosphorylation of Kv1.4, and has similarities to that of phosphorylation of the channel mentioned above.

Second, the influence of synaptotagmin I on the Kv1.4 channel was demonstrated to be Ca^2+^ dose-dependent. In the control experiments, with application of 10 μM BAPTA-AM, the inactivation of Kv1.4 channel was not altered, indicating that the inactivation of Kv1.4 channel itself is Ca^2+^-independent, which is in agreement with the conclusion of Jow et al. [41]. However, when Kv1.4 was coexpressed with synaptotagmin I, the inactivation of Kv1.4 channel was slowed and this change was affected by Ca^2+^ in a dose-dependent manner. These data suggest that synaptotagmin I, functioning as a Ca^2+^ sensor, bestows the calcium sensitivity on the inactivation of Kv1.4 channel.

Third, the impact of synaptotagmin I on Kv1.4 channel is specific. In the brain, A-type currents can be generated by Kv1.4 or any of the subunits in Kv4 family (Kv4.1, Kv4.2 and Kv4.3). Kv1.4 is a presynaptic potassium channel whose mechanism of inactivation is most similar to that of the Kv4 family [18]. In view of this, we examined whether synaptotagmin I affects the Kv4 family subunits that are closely related to the A-type currents. The results (Figure 5) showed that, unlike Kv1.4, Kv4.1, Kv4.2 and Kv4.3 were not significantly affected by synaptotagmin I in terms of the inactivation time constant and the amplitude of channel currents. These results demonstrate that the modulation of Kv1.4 channel by synaptotagmin I has specificity.

Inactivation of Kv1.4 channels is complex, involving at least two distinct mechanisms, N- and C-type inactivation. The N-type inactivation results from the rapid block of the conducting pore by the lipophilic N-terminal region of the channels after channel opening [4], whereas the C-type inactivation occurs through a very distinct mechanism involving closure or collapse of the permeation pathway at both the intracellular and extracellular mouth of the pore [47]. Some auxiliary subunits and other factors can modulate either or both of the N- and C-type inactivation of Kv1.4 alone or in various combinations. For example, coexpression of rat Kvβ1 with Kv1.4 accelerated the N-type inactivation of the channel [39]; Kvβ3 was found to accelerate the N-type inactivation of hKv1.4 [48]. Kvβ2-C and Kvβ2 can enhance N-type inactivation produced by the Kv1.4 α-ball allosterically [42]. It has been suggested that Kvβ1.2 can modulate C-type inactivation in the ferret Kv1.4ΔN2-146 and that is sensitive to external K^+^ concentration [42]. Li et al. [49] reported that extracellular pH modulates both N- and C-type inactivation through an S5-H5 linker histidine. Our experiments demonstrated that, when synaptotagmin I was coexpressed with Kv1.4[K532Y], which had the N-type interaction but limited C-type inactivation, the inactivation of the channels was markedly delayed. So the fourth feature of synaptotagmin I interaction with Kv1.4 is that synaptotagmin I interacts with the N-terminus of Kv1.4 channels to affect the fast N-type inactivation.

### Physiological implications of synaptotagmin I interaction with Kv1.4 channel

Voltage-dependent potassium channels are activated by membrane depolarization and serve to repolarize the membrane [50]. During an action potential spike train, the inactivation of potassium currents can produce progressive broadening of the spikes. Neurons use inactivating K^+^ channels to modulate firing frequency and shape the electrical signaling properties. Slow inactivation delays the repolarization of action potential and attenuates the cell excitability. Kv1.4 channel in neurons has been implicated in the control of action potential frequency, threshold, and shape [51], as well as neurotransmitter release [52,53]. In the present study, synaptotagmin I was demonstrated to interact with the N-terminus of Kv1.4 channel to delay its fast N-terminal inactivation in a Ca^2+^-depedent manner. Although synaptotagmin I did not obviously influence the C-type inactivation of the channel mutant under the present experimental conditions, it can influence the whole inactivation kinetics of the Kv1.4 channel, because there is coupling between N- and C-type inactivated states [19,49,54]. It has been shown that, although N- and C-type inactivation mechanisms are molecularly distinct and subject to independent manipulation, the two inactivation mechanisms strongly interact [55]. The N-type inactivation resulting from the binding of the N-terminal ball to a binding site within the open pore, which likely results in conformation changes of the channel, speeds up the rate at which C-type inactivation occurs [5,54,56]. At the nerve terminal, the delayed inactivation of Kv1.4 channel caused by synaptotagmin I would decrease the action potential frequency, thereby reducing depolarization-dependent Ca^2+^ entry and Ca^2+^-dependent transmitter release. The action of synaptotagmin I on the Kv1.4 channel was speculated to provide a negative feedback mechanism in the regulation of neuronal excitability and synaptic efficacy.

## Materials and methods

### Materials and animals

Trypsin was purchased from Sigma-Aldrich. Plasmid pcDNA3.1 was from Clontech. Protein A-Sepharose beads were from Santa Cruz. Polyclonal antibodies anti-Kv1.4, anti-synaptotagmin I (ab68853) and the peroxidase-conjugated secondary antibodies were from Abcam corporation (HK,China). BAPTA-AM (1,2-bis-(o-Aminophenoxy)- ethane-N,N,N’,N’-tetraacetic acid, tetraacetoxymethyl ester) and DMSO (Dimethyl sulfoxide) was from Sigma– Aldrich Co. (MO, USA). Adult Sprague–Dawley rats (weighting 200–250 g) and HEK293T cells were purchased from the Center South University (Changsha, China).

### Affinity purification of Kv1.4 channel complex

Plasma membrane-enriched fraction was prepared from adult rat hippocampus as previously detailed [57,58], solubilized at 4°C in 1 mg/mL ComplexioLyte48 (incl. protease inhibitors; Logopharm GmbH, Freiburg, Germany) and cleared by ultracentrifugation 30 min at 125, 000 g. 1.0 mL of solubilisate was incubated with 10 μg immobilized rabbit polyclonal Abs raised against Kv1.4 or with control IgGs (Abcam). The bound proteins were eluted with Laemmli buffer and analyzed with SDS-PAGE (DTT was added after elution). The protein bands specifically purified were excised, in-gel digested with trypsin as described previously [59] and subjected to capillary liquid chromatography-tandem mass spectrometry (CapLC-MS/MS). All the experimental procedures involving animals conformed to the guidelines of the National Institutes of Health for the care and use of laboratory animals.

### Mass spectrometry

For CapLC-MS/MS analysis, the digested peptides were injected into a capillary LC system (Agilent 1200) and first desalted and preconcentrated on a pre-column (C18 PepMap™, 0.3 mm i.d., 5 mm long, LC Packings). The outlet of the LC system was directly connected to the electrospray source of an HCTultra™ mass spectrometer (Bruker Daltonics, Germany). Mass spectrometry analysis, data processing and bioinformatics analysis were performed according to our previous work [60]. In the output results, only the bold red peptides with ion scores above 15 in the mascot report were used for protein identification. Proteins were considered to be identified until the first hit of the reverted database appeared. We kept to the principle of using the minimum set of protein sequences to account for all observed peptides. Only proteins with two or more high-confidence (>95%) unique peptides were considered as positively identified.

### Protein expression

Rat Kv1.4, Kv4.1, Kv4.2, Kv4.3 and synaptotagmin I were subcloned into the pCDNA3.1 vector and expressed in HEK293T cells for interaction investigation. In addition, to determine whether synaptotagmin I affects C- or N-type inactivation of Kv1.4, two Kv1.4 cDNA mutants: Kv1.4ΔN (amino acid residues 2–146 deleted to remove N-type inactivation) and Kv1.4[K532Y] (a lysine-tyrosine point mutation introduced to diminish C-type inactivation) were constructed and expressed in the same kind of cells as previously described [5]. The Kv1.4[K532Y] mutation was generated by PCR with the QuikChange mutagenesis method (Stratagene, La Jolla, CA). The fidelity of all clones was verified by sequencing.

### Immunofluorescence microscopy

To investigate the subcellular localization of Kv1.4 and synaptotagmin I, HEK293T cells which were transiently transfected with Kv1.4 or synaptotagmin I cDNA, or cotransfected with both cDNAs were subjected to immunocytochemistry. For immunolocalization of Kv1.4 and synaptotagmin I in rat brain tissue, SD rat (20-day) brain sections (14 μm) were obtained and processed. The primary antibodies and concentration used were rabbit anti-Kv1.4 (Abcam; 1:200) and mouse anti- synaptotagmin I (Millipore, 1:100). Secondary antibodies were applied (1:1 000) as Alexa Fluor 488– or 594–conjugated donkey or goat antibodies to rabbit IgGs or goat antibodies to mouse IgGs, washed extensively in PBS, and mounted with Hoechst 33342 (Beyotime Institute of Biotechnology, China). Controls consisted of omitting the primary antibodies. Images for stained cells were acquired on a Nikon A1R/A1 confocal microscope.

### Cell culture and transfection

HEK293T cells were grown under standard tissue culture conditions (5% CO_2_; 37°C) in Dulbecco’s modified Eagle’s medium (DMEM) supplemented with 10% fetal bovine serum, penicillin (10 g/mL) and streptomycin (10 units/L). Transient transfections were performed via lipofectamine 2000 (Invitrogen, USA). Briefly, cells were seeded directly on 35-mm plastic culture dishes (day 0), allowed to grow to confluency (1–2 days), and then transfected with fresh lipofectamine 2000 (1.5 μg Kv 1.4, 1.5 μg synaptotagmin I, 0.75 μg EGFP, 7 μl lipofectamine 2000). After standing for 4–6 h, the transfection medium was replaced with normal medium. Cells with green fluorescent protein fluorescence were selected for whole-cell patch-clamp recording 36–48 h after transfection.

### Whole-cell patch-clamp recording and data analysis

Kv1.4 potassium currents were recorded on experimental HEK293T cells using whole-cell patch-clamp technique at room temperature (20–25°C). The patch pipettes with DC resistance of 2–3 MΩ when filled with internal solution were fabricated from borosilicate glass tubing (VWR micropipettes,100 μL, VWR Company) using a two-stage vertical microelectrode puller (PC-10, Narishige, Japan) and fire-polished by a heater (Narishige, Japan). Kv1.4 potassium currents were recorded with an Axon 700B patch clamp amplifier (AXON, American) and the P/4 protocol was used to subtract linear capacitive and leakage currents. Ionic currents were sampled at 5 kHz and filtered at 1 kHz. The internal solution contained (in mM): KF 140, EGTA 1, MgCl_2_ 4, and HEPES 10, pH 7.4, and the external bathing solution contained (in mM): NaCl 137, KCl 5.9, MgCl_2_ 1.2, CaCl_2_ 2.2, glucose 14 and HEPES 10, pH 7.4.

CaCl_2_ was added to the intracellular solution in an amount to yield the desired concentration of free Ca^2+^, as calculated with the CABUF program. Currents were elicited by depolarizing steps from -80 to +70 mV in 10 mV increments every 45 s from a holding potential of -80 mV. Data were analyzed by using the program Clampfit10.0 (AXON, American) and ORIGIN 7.0 (Microcal Software, Northampton, MA). Leak subtraction was performed for each trace. Results are expressed as means ± SD. Single exponentials fitted to the decaying phase of current responses to depolarizations at +30 mV were used to obtain the inactivation time constant (τ_inact_). Comparisons between two groups were performed by using Student’s t test. Values of P <0.05 were considered statistically significant.

## Abbreviations

CapLC-MS/MS: Capillary liquid chromatography-tandem mass spectrometry; Kv: Voltage-gated potassium; PCR: Polymerase chain reaction; SDS-PAGE: Sodium dodecyl sulfate polyacrylamide gel electrophoresis; SNARE: Soluble N-ethylmaleimide- sensitive factor attachment proteins receptor; SNAP-25: Synaptosomal-associated protein 25; τinact: Inactivation time constant.

## Competing interests

The authors declare that they have no competing interests.

## Authors’ contributions

XC, WY, CP, WX and LS are responsible for the hypothesis development and overall design of the research and experiment, and supervised the experimental analyses. XC,WX and LS co-wrote the manuscript. XC, SH, GT, YY, PX and CR performed all experiments. All authors read and approved the final manuscript.
